# From Survival to Participation: Early Powered Mobility in the New Era of Spinal Muscular Atrophy Type I

**DOI:** 10.3390/jcm15145673

**Published:** 2026-07-20

**Authors:** Cristina Isabel Díaz-López, Rocío Palomo-Carrión

**Affiliations:** 1 Asociación de Padres de Niños con Dificultades en el Desarrollo APANDID, 45500 Toledo, Spain; 2 Faculty of Physiotherapy and Nursing, Department of Nursing, Physiotherapy and Occupational Therapy, Universidad de Castilla-La Mancha, Avda. Carlos III, s/n, 45071 Toledo, Spain

**Keywords:** spinal muscular atrophy, powered mobility, pediatric rehabilitation participation, International Classification of Functioning, family-centered care, on-time mobility, quality of life, assistive technology, child development

## Abstract

**Background:** Disease-modifying therapies have profoundly changed the natural history of spinal muscular atrophy (SMA) type I, shifting rehabilitation priorities beyond survival and motor function toward participation, autonomy, and quality of life. However, rehabilitation models have not evolved at the same pace, and the role of early powered mobility in this new clinical scenario remains insufficiently conceptualized. **Methods:** This narrative review integrates current evidence on early powered mobility in children with severe motor disabilities with contemporary rehabilitation frameworks, including the International Classification of Functioning, Disability and Health (ICF), participation-based therapy, family-centered care, and the concept of *on-time mobility*. Evidence from the AMEsobreRuedas research program is incorporated to develop a conceptual framework for early powered mobility in children with SMA type I receiving disease-modifying therapies. **Results:** Current evidence suggests that early powered mobility should be understood as a developmental rehabilitation intervention rather than solely as an assistive technology for transportation. Independent mobility facilitates exploration, play, social interaction, autonomy, and participation, while positively influencing family experiences and expectations. Findings from the AMEsobreRuedas program further indicate that the benefits of powered mobility extend beyond driving skill acquisition, supporting participation, quality of life, and family well-being when implemented within meaningful daily contexts. Based on this evidence, a conceptual framework is proposed in which independent mobility acts as an early facilitator of developmental opportunities, with participation emerging through the interaction between the child, family, and environment. **Conclusions:** In the era of disease-modifying therapies, rehabilitation in SMA should move from a motor-centered approach toward a participation-oriented model. Early powered mobility represents a key intervention for promoting developmental opportunities and meaningful participation rather than simply compensating for motor impairment. The proposed conceptual framework may support clinical decision making and provide a foundation for future rehabilitation research in pediatric neuromuscular disorders.

## 1. The Paradigm Shift in Spinal Muscular Atrophy

Spinal muscular atrophy (SMA) is a genetic neuromuscular disease caused by biallelic mutations in the *SMN1* gene, resulting in reduced SMN protein expression and progressive degeneration of alpha motor neurons. This leads to muscle weakness and progressive loss of motor function [1]. Traditionally, SMA type I represented the most severe form of the disease, characterized by early symptom onset, inability to achieve independent sitting, and high mortality during the first years of life in the absence of treatment [1,2].

For decades, clinical management focused on respiratory, nutritional, and orthopedic supportive care aimed at prolonging survival and preserving function, as no treatments were available to modify the natural course of the disease. Consequently, survival and motor progression constituted the main outcome indicators in both clinical practice and research [1,3].

The last decade has represented a turning point in the history of SMA. The approval of disease-modifying therapies, including nusinersen, onasemnogene abeparvovec, and risdiplam, has substantially transformed patient prognosis through different mechanisms aimed at increasing functional SMN protein expression [1,4]. These interventions have demonstrated significant improvements in survival, motor milestone acquisition, and functional stability, particularly when administered early [4]. As a result, a new generation of children with SMA has emerged, presenting functional profiles that differ substantially from those described in the classical natural history and that can no longer be adequately characterized solely by traditional classifications based on age at symptom onset or highest motor milestone achieved [1,4,5].

Nevertheless, these therapeutic advances have not completely eliminated the functional consequences of the disease. Many children continue to experience relevant limitations in independent mobility, autonomy, and participation in daily activities, while previously underexplored dimensions, such as cognitive development, communication, social participation, and quality of life, are becoming increasingly relevant [4,6]. Consequently, rehabilitation goals must now extend beyond survival and motor function to encompass participation, development, and quality of life.

## 2. Methodological Approach

This narrative review aimed to integrate the available empirical evidence and key conceptual frameworks related to early powered mobility in children with spinal muscular atrophy (SMA) type I in the era of disease-modifying therapies, with the objective of proposing a participation-oriented conceptual framework for pediatric rehabilitation.

The literature informing this review was initially identified through a structured bibliographic search conducted during previous research on early powered mobility in children with SMA and other severe neuromuscular disorders. The search was performed in PubMed, Web of Science, CINAHL Complete, the Cochrane Library, ProQuest, and ClinicalTrials.gov using combinations of controlled vocabulary and free-text terms related to spinal muscular atrophy, neuromuscular disorders, pediatric populations, and early powered mobility.

To ensure that the review reflected the most recent evidence, automated literature alerts were maintained in Web of Science and CINAHL Complete throughout manuscript development and were monitored until July 2026. In addition, before submission of the revised manuscript, the original search strategy was rerun in PubMed to identify any newly published studies relevant to the scope of the review.

In addition to empirical studies on early powered mobility, landmark publications addressing child development, the International Classification of Functioning, Disability and Health (ICF), participation-based rehabilitation, family-centered care, and the concept of *on-time mobility* were intentionally incorporated because of their relevance to the development of the proposed conceptual framework.

As a narrative review, studies were not selected according to a predefined systematic review protocol. Instead, the literature was included based on its scientific and conceptual relevance to the objectives of the review, allowing the integration of current evidence within a contemporary rehabilitation paradigm for children with SMA type I.

Accordingly, the purpose of this review was not to systematically summarize all available evidence, but to critically integrate the current literature and contemporary rehabilitation concepts to support the proposed conceptual framework.

## 3. Rehabilitation Must Also Evolve

The changes observed in the clinical course of SMA over the last decade highlight the need to reconsider traditional rehabilitation models. Historically, therapeutic success has been evaluated mainly through indicators related to survival, body function, or the acquisition of specific motor skills [4]. However, this approach is insufficient to capture the complexity of human functioning and the everyday experiences of children with chronic diseases or developmental disabilities.

The publication of the International Classification of Functioning, Disability and Health (ICF) represented an important conceptual shift by moving the focus from disease to functioning [7]. From this perspective, disability does not depend exclusively on the biological characteristics of the individual, but also on the interaction between their abilities, the activities they perform, the participation opportunities available to them, and the personal and environmental factors that shape their context [8].

This shift has important implications for pediatric rehabilitation. If the goal is to improve functioning and participation, interventions should not focus solely on modifying body functions, but also on identifying and addressing factors that facilitate or limit participation. Consequently, the question is no longer only how much a child can move, but to what extent they can participate, explore, and act upon their environment.

In line with this conceptual evolution, intervention models directly aimed at promoting participation in real-life contexts have emerged. Participation-based therapy proposes that the success of an intervention should not be assessed exclusively through changes in body functions or motor performance, but also through the child’s ability to engage in meaningful activities, develop social relationships, and take an active role in everyday life [9]. From this perspective, participation is no longer understood as a secondary consequence of treatment, but as one of its fundamental goals.

In parallel, pediatric rehabilitation has undergone a profound transformation in the way families are understood within the therapeutic process. Caregivers are now recognized as the most stable and influential environment in the child’s life and as possessing unique knowledge of the child’s needs, strengths, and priorities. As a result, families are no longer passive recipients of services, but active collaborators in the planning, implementation, and evaluation of interventions [10,11]. Available evidence supports this approach and highlights that family participation promotes adherence, generalization of learning, and integration of interventions into daily life [12].

In the current context of SMA, where disease-modifying therapies have transformed the functional prognosis of many children, this model is particularly relevant. Beyond traditional motor milestones, outcomes related to participation and everyday functioning need to be incorporated. From this perspective, participation becomes a central goal of contemporary rehabilitation.

## 4. Why Is Self-Initiated Mobility So Important?

Self-initiated mobility is much more than a motor skill. During child development, the ability to move independently allows children to actively explore their environment, access new stimuli, and participate in experiences that promote learning, social interaction, and the progressive acquisition of autonomy [13,14]. Through locomotion, children become less dependent on others to access objects, places, and activities of interest, becoming active agents in their own development.

Available evidence from studies involving typically developing children and children with severe motor disabilities suggests that independent mobility influences multiple developmental domains [15]. Children who acquire autonomous mobility increase their opportunities for exploration, modify their patterns of social interaction, and develop new skills related to communication, cognition, problem-solving, and self-regulation [15,16,17]. In addition, repeated experiences of exploration and control over the environment contribute to the development of self-efficacy and self-determination, which are essential elements for participation throughout childhood [18]. Although disease-specific evidence in SMA remains limited, similar developmental mechanisms are likely to be relevant given the early onset of mobility restrictions.

From this perspective, limitations in autonomous mobility represent not only a motor restriction, but also a restriction in access to opportunities for exploration, learning, and participation [19]. Children with severe motor disabilities often depend on others to interact with their environment, which may reduce personal initiative, limit participation in meaningful activities, and restrict access to experiences that are fundamental for development [20,21].

In response to this issue, the concept of *on-time mobility* has emerged, advocating the provision of independent mobility opportunities during the developmental period in which locomotion plays a key organizing role in development [22,23]. This approach proposes that mobility should not be understood as a reward for achieved development, but as a tool that promotes development. Accordingly, access to independent mobility experiences should not be delayed until the child reaches specific motor or cognitive prerequisites, but should be provided when they are relevant for exploration, learning, and participation [24].

This perspective is particularly relevant for children with early-onset neuromuscular diseases, such as SMA type I, who experience severe restrictions in self-initiated mobility from the first years of life. Although motor limitations may prevent the acquisition of conventional forms of locomotion, the developmental need for exploration, social interaction, and participation emerges at developmental stages similar to those observed in typically developing children. Accordingly, evidence from the broader pediatric rehabilitation literature supports the early introduction of powered mobility opportunities to promote exploration, participation, and development in young children with severe mobility limitations [25,26,27]. These principles are likely to be applicable to children with SMA, although disease-specific evidence remains limited.

## 5. Powered Mobility as a Development-Oriented Intervention

Early powered mobility has emerged as a strategy to provide independent mobility opportunities to children who, due to motor limitations, cannot access conventional forms of locomotion. Its potential does not lie exclusively in compensating for a physical limitation, but in facilitating experiences of exploration, learning, and interaction with the environment during critical stages of development [22,25].

Unlike mobility facilitated by others, self-initiated mobility allows the child to make decisions, direct their own actions, and actively influence the environment [15]. Therefore, powered mobility should be understood as a tool that facilitates participation opportunities, rather than merely as a means of transportation or an assistive device for displacement [26,28].

Available evidence supports this perspective. Several systematic reviews conducted mainly in children with severe motor disabilities rather than specifically in SMA [27,29,30] have described benefits related to environmental exploration, play, social interaction, autonomy, and participation following the introduction of powered mobility devices in children with severe motor disabilities. In addition, these benefits appear to extend to the family environment, fostering more positive expectations regarding children’s abilities and participation opportunities [20,31].

However, the success of these interventions does not depend exclusively on the device used. Participation emerges from the interaction between the child’s abilities, the opportunities provided by the environment, and the support offered by the family. For this reason, contemporary approaches recommend implementing powered mobility programs in natural contexts, integrated into meaningful daily activities, and oriented toward functional and participatory goals [9,27].

Overall, the available evidence supporting early powered mobility is derived from different levels of research. Systematic and scoping reviews involving young children with severe mobility limitations [29,30], together with clinical studies conducted in heterogeneous pediatric populations [31,32], consistently support the developmental benefits of early powered mobility and its role as a development-oriented intervention. However, disease-specific evidence in children with SMA type I remains limited, with only a small number of studies directly evaluating this population [33].

## 6. Lessons Learned from the AMEsobreRuedas Program

The developmental principles discussed above are largely supported by evidence from broader pediatric rehabilitation populations. Building upon this evidence, the following section focuses specifically on findings generated in children with SMA type I through the AMEsobreRuedas research program. In addition to the previously published literature, this research program has provided novel clinical insights that may help guide the design of future powered mobility interventions in this population.

### 6.1. From Learning to Drive to Participating: Mobility Becomes Meaningful When It Has a Functional Purpose

One of the principal findings of the AMEsobreRuedas program is that children with SMA type I can acquire powered mobility skills more rapidly and effectively than traditionally assumed [34]. Progression assessed through the Assessment of Learning Powered Mobility Use (ALP) showed significant improvements from the first weeks of training, regardless of chronological age or initial level of device-use learning. Most participants reached advanced learning levels during the intervention period, suggesting that the ability to use a powered mobility device functionally does not depend exclusively on the presence of specific prior motor skills [34].

These findings support the principles of *on-time mobility*, according to which mobility opportunities should be provided when they are developmentally relevant, rather than when the child proves to be ready for them [22,23]. They also highlight the importance of individualized adaptations. Postural modifications, alternative access systems, and device-specific adjustments enabled children with very different functional profiles to actively participate in training from the earliest stages [35].

However, perhaps the most relevant finding was not that children learned to drive a device, but how they used this ability. The functional goals achieved showed that mobility became meaningful when it served as a tool to access activities relevant to the child and family. Participants used mobility to approach other people, access toys, transport objects, participate in leisure activities, and explore spaces autonomously. In this sense, movement was rarely an end in itself; rather, it became a means to support participation, autonomy, and interaction with the environment [34].

This observation also identified an important area for improvement in future interventions. Although training sessions conducted in community contexts exposed children to different real-life scenarios, many activities continued to focus on practicing driving and displacement skills. Future interventions may therefore benefit from a more explicit planning of functional and participatory goals, progressively shifting the focus from learning to drive toward using mobility to engage in meaningful activities in everyday life.

From this perspective, the success of an intervention should not be measured solely by the child’s ability to control a device, but by the opportunities that mobility creates for exploring, playing, interacting, making choices, and participating in activities that are relevant to daily life. This approach is consistent with contemporary participation-centered rehabilitation models and represents one of the main lessons learned from the AMEsobreRuedas program.

### 6.2. Participation Depends on Both Mobility and Context

Another relevant finding from the AMEsobreRuedas program was that changes in participation were not distributed homogeneously across everyday contexts [36]. Although powered mobility expands opportunities for action, exploration, and interaction, the extent to which these opportunities are transformed into real participation experiences depends largely on the characteristics of the environment in which the activity takes place.

The results showed more consistent improvements in the home environment than in the community [36]. This finding suggests that access to independent mobility is a necessary condition for participation, but not sufficient to guarantee participation across all contexts. Previous participation-based rehabilitation literature and evidence syntheses in children with severe mobility limitations [9,27] similarly emphasize that participation depends not only on the child’s abilities but also on environmental opportunities and contextual support. Whereas the home usually provides a relatively flexible environment that can be adapted to the child’s needs, the community introduces additional demands related to physical accessibility, the complexity of social interactions, and environmental barriers beyond family control [9,27,36].

Complementarily, the program findings also showed that improvements in social-cognitive skills were associated with better quality of life [37], whereas powered mobility learning was related to greater home participation [36]. Taken together, these findings suggest that powered mobility may influence different dimensions of child functioning, although still incompletely understood, mechanisms.

From a clinical perspective, these findings invite a reconsideration of how powered mobility interventions are designed. Previous literature on powered mobility learning, primarily involving children with severe motor disabilities rather than specifically children with SMA [38,39] has traditionally focused on facilitating device access and the acquisition of driving skills. However, the AMEsobreRuedas findings suggest that it is also necessary to intervene in the contexts where participation occurs, facilitating real opportunities for engagement in meaningful activities and addressing environmental barriers that limit the functional use of mobility in everyday life.

### 6.3. Mobility Transforms Family Experiences and Expectations of Participation

Another major lesson learned from the AMEsobreRuedas program was that the effects of powered mobility were not limited to children, but also had a significant impact on the experiences and perceptions of their families. Quantitative findings showed a reduction in parental stress throughout the intervention, suggesting that access to new opportunities for autonomy and participation may positively influence family well-being [37].

Qualitative findings helped to better understand the mechanisms that may explain these changes. Families described a progressive transformation in how they perceived their children’s abilities, broader expectations regarding their potential for participation, and greater confidence in their possibilities for interaction with the environment [40]. In this sense, the perceived benefits were not related only to the acquisition of driving skills, but to the opportunities that mobility created for exploring spaces, making choices, participating in daily activities, and interacting with others.

Similarly, powered mobility was frequently described as a tool that enabled the child to take a more active role within family dynamics, supporting experiences of autonomy that had previously been difficult to achieve [25]. As a result, families tended to progressively shift their focus from disease-related limitations toward their children’s abilities, interests, and participation possibilities [40,41].

These findings further support the active involvement of families during powered mobility implementation, as discussed in the following section.

## 7. Clinical Considerations for Implementing Early Powered Mobility

### 7.1. Candidate Selection and Optimal Timing

Early powered mobility should be considered for children with SMA who are expected to experience significant limitations in self-initiated mobility despite current disease-modifying therapies [4,9]. Rather than relying exclusively on chronological age or specific motor milestones, candidate selection may be guided by the child’s developmental need for independent exploration, play, participation, and interaction with the environment [13,14].

Consistent with the concept of *on-time mobility*, powered mobility opportunities should ideally be introduced during the developmental period in which typically developing children begin to acquire independent locomotion, thereby minimizing the risk of prolonged mobility deprivation during a critical period of development [22,23]. Nevertheless, clinical decision making should remain individualized, taking into account the child’s functional abilities, medical stability, motivation, family priorities, and environmental context [25,27].

### 7.2. Family-Centered Implementation

Successful implementation of early powered mobility extends beyond providing access to a powered mobility device [40]. Consistent with contemporary family-centered rehabilitation approaches, intervention should actively involve caregivers as partners throughout the rehabilitation process [7,8,10]. Families play a fundamental role in creating meaningful opportunities for practice within everyday routines, facilitating participation in natural environments, and integrating powered mobility into daily life [40,42]. Emerging evidence also suggests that caregiver expectations and perceptions of their child’s capabilities may influence powered mobility learning, reinforcing the importance of actively involving families throughout the intervention process [42].

Regular professional follow-up is also essential to support families, establish shared functional goals, adapt intervention strategies as the child’s needs evolve, and build caregiver confidence throughout the learning process [34,40,43]. Rather than relying on isolated training sessions, frequent opportunities for practice integrated into meaningful daily routines, supported by regular professional coaching and active family involvement, are likely to facilitate motor learning and promote participation in everyday activities [40,43]. This collaborative approach may involve physiotherapists, occupational therapists, and other professionals according to local resources and healthcare organization.

### 7.3. Practical Barriers and Facilitators

Successful implementation of early powered mobility depends on multiple interacting factors beyond the child’s motor abilities, reflecting the interaction between the child, the environment, and the opportunities for participation described in the ICF framework [7,9]. Environmental accessibility and opportunities for practice within meaningful natural environments are key determinants of successful participation and long-term use of powered mobility [25,27,30]. In addition, limited availability of powered mobility devices and funding restrictions may represent important barriers to early access to powered mobility [27,44]. Family engagement and caregiver training also play a central role in successful implementation, as they facilitate opportunities for practice, support children’s learning, and influence long-term use of powered mobility [40,42].

Importantly, the proposed conceptual framework should not be interpreted as being linked to a specific powered mobility device. Although the AMEsobreRuedas program employed modified electric ride-on toy cars because of their adaptability, accessibility [35], the developmental principles described in this review are not dependent on a particular technology. Rather, any device that provides adequate postural support, safety and independent access to the control interface may fulfil the same rehabilitative purpose. Recent studies further suggest that low-cost powered mobility devices may represent an effective and accessible strategy for introducing early independent mobility within pediatric rehabilitation programs [35,44]. Modified ride-on toy cars and other low-cost powered mobility devices may represent attractive options for introducing independent mobility during early childhood because they are substantially less expensive than conventional powered wheelchairs, can often be adapted using simple modifications, and have shown promising feasibility, participation, and satisfaction outcomes in recent clinical studies [35,44].

Finally, although early powered mobility offers important developmental opportunities, it may not be equally feasible or appropriate for every child with SMA. Decisions regarding its implementation should remain individualized and consider the child’s clinical condition, functional abilities, capacity to engage with the intervention, family preferences, and environmental circumstances.

## 8. From Assistive Technology to Rehabilitation Intervention

### 8.1. Mobility as a Facilitator of Developmental Opportunities

The results of AMEsobreRuedas suggest a possible sequence of effects in which mobility acts as an intermediate facilitator of development [36,37]. Initially, children acquire skills related to device control and autonomous displacement. These skills then begin to transfer to functional activities and everyday contexts, providing access to new experiences of exploration, play, and social interaction. As these opportunities become integrated into daily life, changes may occur in broader domains related to function, participation, quality of life, and family well-being [37].

From this perspective, mobility does not constitute the final outcome of the rehabilitation process, but rather the mechanism that enables access to experiences capable of promoting development and participation. Its value does not lie solely in enabling displacement, but in expanding the opportunities available for the child to act upon the environment and actively participate in it.

### 8.2. Proposed Conceptual Framework for SMA in the Era of Disease-Modifying Therapies

**Figure 1:**
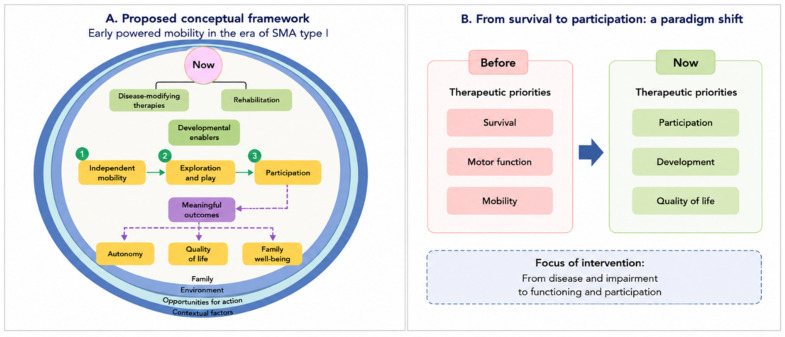
Conceptual framework for early powered mobility in spinal muscular atrophy in the era of disease-modifying therapies. (**A**) Proposed hypothesis-generating conceptual framework based on the currently available evidence and the findings of the AMEsobreRuedas research program. Independent mobility may facilitate exploration and play experiences, which in turn may promote participation. Green arrows indicate relationships supported by the available evidence, whereas purple arrows represent conceptual relationships proposed by the authors to illustrate potential pathways linking participation with meaningful child and family outcomes, including autonomy, quality of life, and family well-being. The entire process is modulated by contextual factors. (**B**) Representation of the paradigm shift in SMA from a traditional approach focused on survival, motor function, and mobility toward a contemporary model centered on development, participation, and quality of life. This panel illustrates the evolution of rehabilitation priorities rather than a causal pathway.

Based on the currently available evidence and the findings generated through the AMEsobreRuedas research program, we propose a conceptual framework in which independent mobility may represent an early facilitator of exploration and play experiences [34]. These experiences may create opportunities for interaction and participation that could, in turn, contribute to meaningful outcomes for the child and family, including autonomy, quality of life, and family well-being [37]. Rather than representing an established causal pathway, this framework is intended to integrate the available evidence, illustrate potential relationships between these domains, and guide future research. Throughout this process, outcomes are likely to be influenced by contextual factors, particularly the family, the environment, and the available opportunities for action [40]. The proposed conceptual framework is illustrated in Figure 1A.

This framework is situated within a broader paradigm shift currently taking place in SMA rehabilitation (Figure 1B). Whereas therapeutic priorities historically focused on survival, motor function, and mobility, the era of disease-modifying therapies has progressively shifted the focus toward development, participation, and quality of life. From this perspective, early powered mobility should not be viewed exclusively as an assistive technology, but as a rehabilitation intervention aimed at generating opportunities for development and participation.

The novelty of the proposed framework therefore lies not in introducing new rehabilitation concepts, but in bringing together established theoretical principles within a disease-specific model that places early powered mobility as a potential developmental rehabilitation intervention for children with SMA type I.

## 9. Clinical Implications and Future Research Directions

### 9.1. Implications for Clinical Practice

Viewing powered mobility as a development-oriented intervention has several implications for clinical practice. First, its implementation should not depend exclusively on the prior acquisition of specific motor or cognitive skills [24,34], but rather on the early identification of situations in which mobility limitations may compromise the child’s development, independence, participation, or quality of life. This approach is consistent with the principles of *on-time mobility*, which advocate providing independent mobility experiences at the developmental stage when they are most relevant [22,23].

The AMEsobreRuedas findings also suggest that interventions should be designed around specific functional and participatory goals, rather than focusing exclusively on the acquisition of driving skills [34]. Integrating mobility into meaningful activities related to play, family routines, and community participation may support a more effective transfer of acquired skills into everyday life [40].

Finally, the implementation of powered mobility programs requires an ecological and family-centered approach that simultaneously considers the child’s needs, the characteristics of the environment, and the real participation opportunities available in daily life.

### 9.2. Future Research Directions

Despite growing interest in early powered mobility, important knowledge gaps remain regarding methodological heterogeneity, inconsistent intervention protocols, small sample sizes, limited measurement of intervention fidelity, and the short duration of follow-up in available studies [30,31,32,33,45]. In addition, future studies should also clearly define the core components of powered mobility interventions and systematically evaluate intervention fidelity to improve transparency, reproducibility, and implementation across different rehabilitation settings [45].

Future studies should move beyond evaluating powered mobility skills alone and adopt standardized outcome measures that capture the broader developmental impact of early powered mobility interventions. These measures should include participation, functional performance, quality of life, caregiver outcomes (e.g., caregiver burden, stress, and family well-being), and long-term developmental outcomes. Such standardization would facilitate comparisons across studies and strengthen future evidence syntheses and meta-analyses.

Although the AMEsobreRuedas research program has generated one of the largest disease-specific research programs currently available on early powered mobility in children with SMA type I, independent replication in different healthcare systems, rehabilitation settings, and research groups will be essential to determine the generalizability and external validity of the proposed conceptual framework and to refine its application across diverse clinical contexts.

Further research is also needed to explore strategies to optimize participation in community contexts and to evaluate training programs specifically oriented toward participatory goals.

Finally, the emergence of new clinical profiles resulting from early treatment and newborn screening highlights the need to explore the role of independent mobility in the development and participation of this new generation of children with SMA.

## 10. Conclusions

The emergence of disease-modifying therapies has transformed the goals of rehabilitation in SMA. In this new scenario, early powered mobility should not be understood solely as an assistive technology aimed at compensating for motor limitations, but as a rehabilitation intervention capable of generating opportunities for exploration, participation, and development. Ultimately, the value of early powered mobility lies not in the device itself, but in the developmental opportunities it creates for children and their families.

## Data Availability

No new data were generated in this study. All data supporting the findings of this review are available in the published literature cited in the reference list.
